# The effect of whole-body electromyostimulation on visceral adipose tissue volume in overweight-to-obese adults with knee osteoarthritis: A randomized controlled study

**DOI:** 10.3389/fphys.2025.1544332

**Published:** 2025-04-15

**Authors:** Benazir Burkhardt, Oliver Chaudry, Stephanie Kast, Simon von Stengel, Matthias Kohl, Frank W. Roemer, Klaus Engelke, Michael Uder, Wolfgang Kemmler

**Affiliations:** ^1^ Institute of Radiology, University Hospital Erlangen, Erlangen, Germany; ^2^ Institute of Medical Physics, Friedrich-Alexander University Erlangen-Nürnberg, Erlangen, Germany; ^3^ Department of Medical and Life Sciences, University of Furtwangen, Schwenningen, Germany; ^4^ Department of Medicine III, University Hospital Erlangen, Erlangen, Germany

**Keywords:** electromyostimulation, exercise, intra-abdominal fat, visceral fat, magnetic resonance imaging, obesity, adults

## Abstract

**Introduction:**

Physical exercise favorably affects visceral adipose tissue (VAT), which is a risk factor for cardiometabolic diseases. However, many people are unable or unwilling to conduct frequent and intensive exercise programs that have favorable effects on VAT. The present study aimed to determine the effect of time-efficient and joint-friendly whole-body electromyostimulation (WB-EMS) technology on VAT volume in overweight-to-obese adults with osteoarthritis of the knee.

**Methods:**

In total, 46 women and 26 men (58.4 ± 7.0 years; BMI: 30.2 ± 4.2 kg/m^2^) with femuro-tibial knee osteoarthritis were randomly allocated to WB-EMS (n = 36) with 1.5 × 20 min/week for 29 weeks or a usual care control group (CG: n = 36) with six sessions of physiotherapy. Magnetic resonance imaging (MRI) using a non-contrast enhanced two-point Dixon gradient echo volumetric interpolated breath-hold examination determined the VAT from mid L2 to mid L3.

**Results:**

In summary, VAT volume increased non-significantly in the CG (p = 0.246) and decreased non-significantly in the WB-EMS group (p = 0.143). We failed to determine significant WB-EMS-induced effects, i.e., group differences for absolute changes in the VAT volume (p = 0.090). However, we observed gender differences with significantly higher effects in men than in women (p = 0.032).

**Discussion:**

We conclude that low volume, non-superimposed WB-EMS is not a perfect tool for decreasing VAT, particularly in overweight-to-obese women.

## 1 Introduction

Visceral adipose tissue (VAT) is a complex and metabolically active tissue that produces different adipokines and hormones (Silveira et al., 2021). Accumulation of VAT is a strong predictor of all-cause, cardiovascular- and cancer-specific mortality (Brown et al., 2016). In addition, many studies have provided evidence that VAT is closely related to an increased risk of insulin resistance (Kim and Kim, 2023; Zhang et al., 2015), atherosclerosis (ohman et al., 2009), dyslipidemia (von Kruchten et al., 2022), hypertension (Hall et al., 2015), hepatic steatosis (Park et al., 2008), coronary heart disease (Chen et al., 2022; Silveira et al., 2021), and different types of cancer (Silveira et al., 2021). There is striking evidence that participating in exercise interventions had favorable effects on accumulated VAT (Chen et al., 2023; Lee and Lee, 2021). In summary, combined aerobic and resistance exercise seems to be particularly effective in reducing abdominal obesity in overweight/obese individuals (Batrakoulis et al., 2022). However, considering the time required for these protocols (≈2–3 × 30–45 min), many people, with or without overweight and obesity, might be unmotivated or unable to join frequent, time-consuming exercise programs. Similar to the popular high-intensity interval training (HIIT) (A'Naja et al., 2024), whole-body electromyostimulation (WB-EMS) has been recognized as a very time-efficient training technology (Kemmler et al., 2020) to address cardiometabolic conditions (Batrakoulis et al., 2021; Guretzki et al., 2024). However, in contrast to HIIT, WB-EMS is much more joint-friendly, which may particularly attract people with joint disorders. Unfortunately, there is limited evidence of the positive effects of WB-EMS on VAT. A recent study provided significant positive evidence for the WB-EMS effects on intraabdominal fat (Park et al., 2021) in obese elderly women; however, the authors applied a rather unusual (Beier et al., 2024), high-volume approach with aerobic dance superimposed by WB-EMS. As this approach differs greatly from the low-volume, non-superimposed concepts primarily applied in research (Beier et al., 2024) and commercial WB-EMS settings (Kemmler et al., 2024), the aim of the present study was to determine the effect of a time-effective, joint-friendly WB-EMS program on VAT in overweight or obese people. Since there is strong evidence that the VAT response to RT exercise is more pronounced in men (Chen et al., 2023), we also considered gender differences in our analyses of VAT changes, and we hypothesized that the low-volume, non-superimposed WB-EMS program significantly decreases VAT compared to the control group.

## 2 Material and methods

### 2.1 Study design

The randomized controlled WB-EMS trial (RCT) is part of the “electromyostimulation for the treatment of knee osteoarthritis (OA) (EMSOAT) study.” This study applied a balanced parallel group design (WB-EMS versus control group) and evaluated the effects of 7 months of WB-EMS application on outcomes related to knee osteoarthritis in middle-aged and older adults who were overweight and obese. The present study focused on WB-EMS effects on visceral adipose tissue volume. Briefly, the EMSOAT was planned, initiated, and conducted by the Institute of Radiology, University Hospital Nürnberg, Germany. The University Ethics Committee of the FAU approved this trial (No. 352_20 B), which fully complies with the Helsinki Declaration “Ethical Principles for Medical Research Involving Human Subjects”(World_Medical_Association, 2013). After receiving detailed information, all the study participants provided their written informed consent. This project was fully registered under ClinicalTrials.gov (NCT05672264).

#### 2.1.1 Participants

**FIGURE 1:**
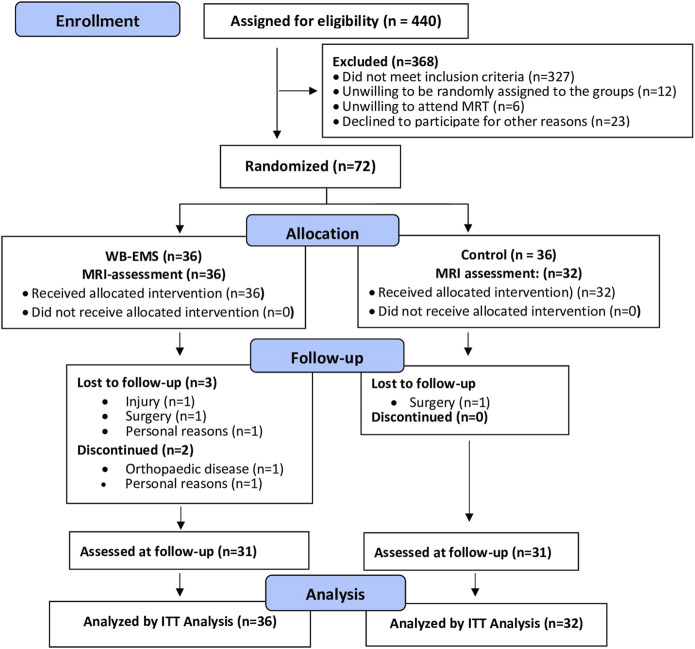
Participant flow throughout the study adapted for MRI assessment.

Briefly, between March 2022 and June 2022, local newspapers, social media, and selected physicians disseminated our study calls, which included the core eligibility criteria. A total of 440 women and men responded via email or telephone and were provided with more detailed written study information. Potential participants who confirmed their preliminary eligibility were further checked through detailed standardized phone calls conducted by carefully briefed research assistants. Inclusion criteria were (a) age 40–70 years old; (b) overweight or obese (BMI>25 kg/m^2^); (c) fulfilling clinical ACR criteria for knee OA (Altman et al., 1991); and(d) osteoarthritic knee pain on at least 50% of the days during the last 3 months with an average pain intensity of >2.5 on a 0–10 numerical rating scale (Bennell et al., 2020). Exclusion criteria were (a) any WB-EMS application or regular resistance exercise (≥60 min per week) during the last 12 months; (b) glucocorticoid or opioid pain therapy; (c) trauma of the knee joint within the last 12 weeks; (d) intra-articular injections in the knee joint in the last 12 weeks; (e) conditions, diseases, and corresponding therapy with a relevant impact on our study outcomes (e.g., rheumatoid arthritis and fibromyalgia); (f) contraindications for WB-EMS (Kemmler et al., 2019) or MRI application; and (g) ≥4 weeks of absence during the 29-week conditioning and intervention phase. Lastly, the study physician verified the final eligibility. Twelve of the 84 eligible participants refused to participate predominately due to the random allocation to the study groups (WB-EMS or control). Thus, finally 72 participants were eligible and willing to participate in the present study. However, mainly due to time constraints, four participants (all CG) were unable to join the MRI assessments (i.e., baseline and 29-week control); thus, 68 participants (43 women and 25 men) with MRI data were included in the present analysis of the WB-EMS effects on visceral adipose tissue changes (Figure 1; Table 1).

**TABLE 1:** Baseline characteristics of the study participants.

Variable	CG (n = 32) MV ± SD	WB-EMS (n = 36) MV ± SD	p
Gender (women/men) [n]	21/11	22/14	0.803
Age [years]	59.2 ± 6.0	58.3 ± 7.2	0.602
Body height [cm]	174.3 ± 9.5	173.2 ± 9.9	0.628
Body height, men [cm] Body height, women [cm]	183.5 ± 7.2 169.6 ± 6.6	183.4 ± 6.7 166.7 ± 4.8	0.993 0.107
Body mass [kg]	89.4 ± 15.7	93.2 ± 15.1	0.322
Body mass, men [kg] Body mass, women [kg]	101.3 ± 14.2 83.2 ± 12.8	102.5 ± 13.7 87.2 ± 13.0	0.832 0.315
Lean body mass (LBM) [kg]^a^	57.8 ± 12.4	60.2 ± 12.5	0.435
LBM men [kg] LBM women [kg]	71.1 ± 10.5 50.9 ± 6.1	73.9 ± 7.4 51.5 ± 4.5	0.429 0.730
Total body fat [%]^a^	35.3 ± 7.4	35.2 ± 9.2	0.946
Total body fat, men [kg] Total body fat, women [kg]	29.6 ± 6.4 38.2 ± 6.2	27.3 ± 5.9 40.1 ± 7.4	0.358 0.373
Waist circumference [cm]	101.3 ± 11.9	102.7 ± 10.9	0.616
WC men [cm] WC women [cm]	108.7 ± 8.2 97.4 ± 11.8	109.5 ± 8.4 98.3 ± 10.0	0.801 0.790
Obesity (BMI ≥30.0 kg/m^b^) [n]^b^	17	18	0.814
Physical activity [index]^b^	3.6 ± 1.1	3.6 ± 1.3	0.878
No frequent exercise (≥60 min/w) [n]^b^	18	18	0.606
Mean arterial pressure [mmHg]	104.8 ± 9.5	103.6 ± 9.4	0.536
Number of diseases [n]^b^	1.47 ± 1.11	1.31 ± 0.95	0.515
Number of medications [n]^b^	1.25 ± 1.19	1.22 ± 1.27	0.926

^a^ As assessed by bioimpedance analysis (BIA).

^b^ As assessed by detailed questionnaires.

#### 2.1.2 Randomization and blinding

Participants allocated themselves to the WB-EMS or control group by drawing lots. Lots were placed in small opaque capsules (“kinder egg”, Ferrero, Italy) and drawn from a bowl. A researcher who was not involved in the present project prepared the lots to ensure allocation concealment. After the randomization procedure, the primary investigator (SK) enrolled participants and instructed them in detail about their study status and corresponding dos and don´ts. Research assistants, testers, and outcome assessors were unaware of the participants’ group status (WB-EMS or CG) and were not allowed to ask either.

### 2.2 Study procedures

The WB-EMS training group (WB-EMS) conducted 7 months of WB-EMS application as described below, while the control group (CG) was provided with a “usual care” intervention (physiotherapy – see below). Furthermore, the WB-EMS and CG completed a self-management education program for knee OA. Apart from the intervention described above, participants were instructed to maintain their lifestyle, including physical activity, exercise, and dietary habits during the intervention period. There was a monthly reminder about this requirement during the self-education sessions.

#### 2.2.1 WB-EMS intervention

**FIGURE 2:**
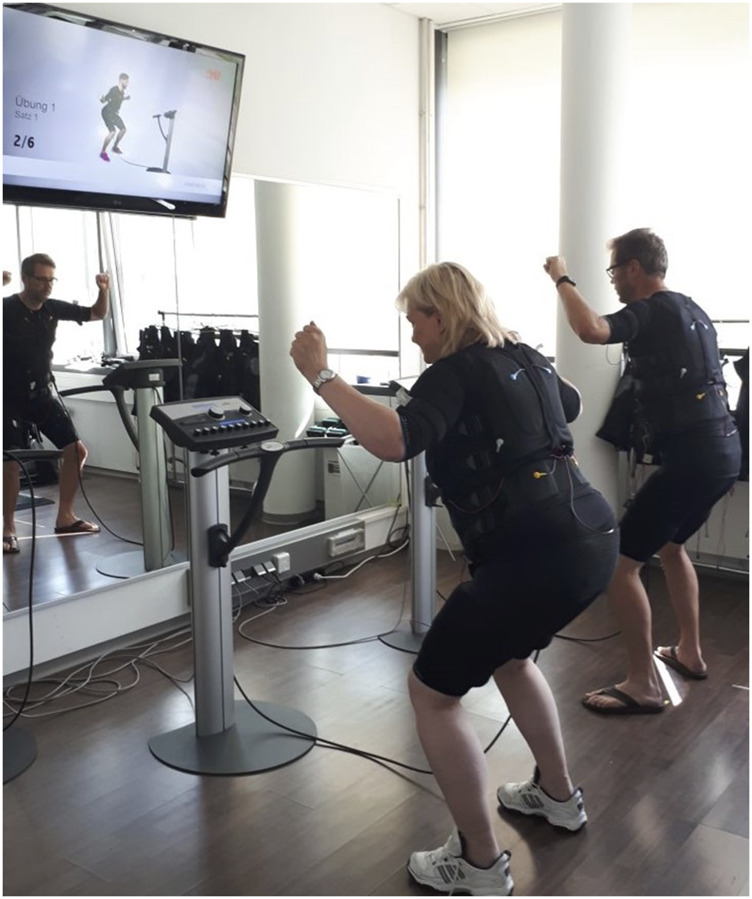
Example of a typical exercise/movement performed during the WB-EMS session.

We conducted a standard WB-EMS session (Beier et al., 2024; Kemmler et al., 2021) 1.5 × 20 min per week (e.g., every Monday and every second Thursday) for 29 weeks, including 4 weeks of familiarization and conditioning with the Miha Bodytec type II device (Gersthofen, Germany). Briefly, the thighs and upper arms, gluteals, abdomen, chest, lower back, latissimus, and upper back were stimulated simultaneously but with dedicated impulse intensity. Using bipolar current, the WB-EMS protocol scheduled an impulse low frequency of 85 Hz, an impulse width of 350 µs, and a direct impulse boost with 6 s of EMS stimulation intermitted by a 4 s impulse break. Based on the close interaction between the licensed trainer and a maximum of two trainees, the impulse intensity as prescribed by the rate of perceived exertion (RPE) was carefully increased to “6-7” (i.e., “hard+ to very hard”) on the Borg CR10 Scale (Borg, 2010). During the session, the impulse intensity for all electrodes was carefully adjusted every 3 min to ensure a constant impulse intensity (Kemmler et al., 2023). Video-guided low-intensity exercises (low-amplitude squat with latissimus pulleys, butterfly reverse with angled arms, straight pullovers with trunk flexion, standing trunk flexion, one-legged stand with biceps curl, side step with weight shift, and biceps curl (Weissenfels et al., 2019)) without additional load were conducted during the 6s impulse phase in a standing position. Figure 2 shows an example of a typical exercise/movement conducted during a WB-EMS session.

#### 2.2.2 Control intervention (physiotherapy)

As per the standard care practice in Germany, the control group was provided with six standardized physiotherapy sessions of 20 min each. Physiotherapy treatment was carried out individually in the sense of “standard care” in a diagnosis-oriented manner. The specific content was at the discretion of the treating physiotherapists and included techniques and exercises for reducing pain and detonization of the muscle tissue, increasing mobility of the knee joint, and strengthening leg muscles.

#### 2.2.3 Self-management

A self-management program for osteoarthritis (Nelson et al., 2014) with six sessions of 60 min each was applied for both groups. Briefly, the program aimed to provide education, information, and counseling to prevent the progression of OA, reduce fear and avoidance attitudes, and improve the quality of life and mobility of the participants.

### 2.3 Study outcomes

As stated, the EMSOAT study predominately focuses on outcomes related to knee osteoarthritis. The present study addressed abdominal fat changes considered secondary outcomes of the EMSOAT project.

• Visceral adipose tissue (VAT) volume between baseline and 29-week follow-up (FU)

#### 2.3.1 Explanatory outcomes

• Changes in the physical activity and exercise between baseline and 29-week FU
• Changes in the dietary intake between baseline and 29-week FU
• Changes in the medication use between baseline and 29-week FU.

### 2.4 Assessments

#### 2.4.1 MRI data acquisition and examination

**FIGURE 3:**
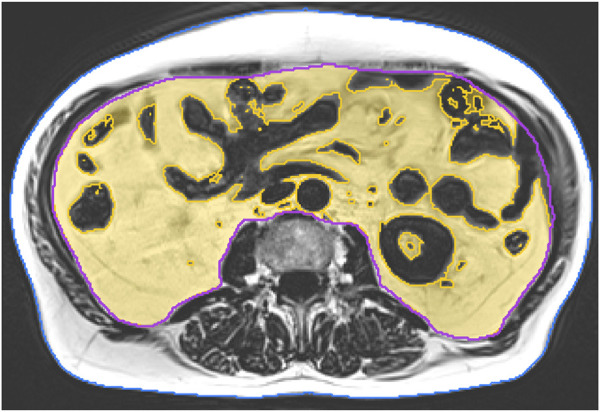
Final segmentation result of VAT (yellow overlay) without inner organs and intestines, inner abdominal volume (magenta contour), and outer contour of the body (blue)”.

MRI scans were acquired at baseline and after 29 weeks of intervention. All scans were consistently acquired using a 3 T scanner (PRISMA, Siemens Healthineers, Erlangen, Germany). We applied a non-contrast-enhanced two-point Dixon Gradient Echo Volumetric Interpolated Breath-hold Examination (VIBE) sequence (TE: 1.29 ms; TR: 3.97 ms; matrix size: 320 × 260; voxel size: 1.2 × 1.2 × 3.5 mm^3^; and slice gap: 0.7 mm). Twelve slices covered a total length of approximately 5 cm from mid-L2 to mid-L3. Image analysis was performed using the medical image analysis framework (MIAF, Friedrich-Alexander University Erlangen-Nürnberg), as described in detail in a previous publication (Chaudry et al., 2020). The first and last slices were excluded because of intensity inhomogeneity. In the remaining ten slices, the outer contour of the body was determined automatically. The contour of the abdominal cavity was manually segmented by a supervised and trained research assistant. This was performed slice by slice using the Fiji open-source software (Schindelin et al., 2012). Between the measurements of each participant, the position of the scanned volumes was evaluated, and non-overlapping slices were cut off if necessary. In order to separate the VAT inside the abdominal cavity from the inner organs such as the kidneys, intestines, and blood vessels, we used a threshold calculated using the Otsu method (Otsu, 1979) (Figure 3).

#### 2.4.2 Baseline characteristics and confounding factors

Body height was assessed using a Holtain stadiometer (Crymych Dyfed, United Kingdom). Direct-segmental multi-frequency bioimpedance analysis (DSM-BIA, InBody 770, Seoul, Korea) was used to determine the body mass and body composition. Overweight (25.0–29.9 kg/m^2^) and (≥30.0 kg/m^2^) obesity were classified according to the body mass index (BMI).

At baseline, a detailed standardized questionnaire collected information on (a) demographic parameters; (b) physical limitations, diseases, operations, pharmacologic therapy, dietary supplements; and (c) lifestyle, including physical activity, exercise, and diet. After 29 weeks of intervention, all participants completed the FU questionnaire that aimed to determine changes in conditions/diseases, pharmacologic and physical therapy, physical activity, exercise, and diet, i.e., factors with a potential impact on the present outcomes. The questionnaires were carefully checked for consistency, completeness, and accuracy by the primary investigator (SK), together with the participants.

### 2.5 Sample size calculation

The sample size calculation was based on a parameter not addressed in the present study. Briefly, 36 participants per group were needed to determine the effects on the primary outcome, “pain of the knee joint,” as determined by the KOOS (Knee Injury and Osteoarthritis Outcome Score; (Roos and Lohmander, 2003)) questionnaire, applying a statistical power of 80%, an α-level of 5%, and a two-tailed t-test approach.

### 2.6 Statistical analysis

As recommended for RCTs, we applied the intention-to-treat (ITT) principle that included all participants randomly assigned to the study arms (WB-EMS vs CG), regardless of their loss to follow-up. However, as reported, due to the lack of baseline MRI and FU data, four participants in the CG could not be included in the analysis. We used R statistics software (R Development Core Team Vienna, Austria) in combination with Amelia II (Honaker et al., 2011) for multiple imputation (ITT). The full data set was used for multiple imputations, with the imputation repeated 100 times. Normal distribution was checked graphically (gg-plots and residual plots). Within-group changes were analyzed using the t-test. ANCOVA adjusted for baseline differences in VAT volume was applied to determine between-group differences (i.e., “effects”) after 29 weeks. A second ANCOVA was adjusted for baseline VAT volume and gender to determine possible gender differences. Differences in the distribution of categorical variables were analyzed using Pearson’s chi-square tests (Table 1). The standardized mean difference (SMD) was calculated using Cohen (Cohen’s d). All tests were 2-tailed, and significance was set at p < 0.05.

## 3 Results

Table 1 displays the baseline results of 43 women and 15 men (58.7 ± 6.9 years; BMI: 30.2 ± 4.1 kg/m^2^) with MRI data. In summary, no significant differences were observed between the WB-EMS and the CG at baseline.

Drop-out and loss to follow-up are displayed in Figure 1. Briefly, four participants (WB-EMS: n = 3 vs CG: n = 1) were unable to attend the 29-week FU assessment. A further two participants of the WB-EMS group quit the study due to reasons not related to the intervention. The attendance rate averaged 88% ± 10% in the WB-EMS group. Predominately due to diseases, four participants (WB-EMS) exercised, on average, less than once a week. Attendance in the physiotherapy sessions (CG) was >90%. Adherence to the WB-EMS or physiotherapy (CG) protocol did not differ significantly between the genders. Furthermore, no adverse effects or injuries were observed during the WB-EMS sessions, and (apart from occasional delayed onset muscular soreness) no participants reported any WB-EMS-related relevant discomfort during or after WB-EMS application.

### 3.1 Study outcome

Table 2 shows the results for VAT at baseline and the changes after 29 weeks of intervention adjusted for baseline VAT (ANCOVA). In summary, no significant WB-EMS-induced effects (p = 0.090; d’ = 0.43), i.e., group differences for absolute changes between WB-EMS and CG, on the visceral adipose tissue volume were observed. In detail, VAT volume increased non-significantly in the CG (p = 0.246) and decreased non-significantly in the WB-EMS groups (p = 0.143).

**TABLE 2:** Baseline data and changes in visceral adipose tissue parameters in the CG and WB-EMS groups. SMD: standardized mean difference;^n.s.^: non-significant intra-group changes.

	CG (n = 32) MV ± SD	WB-EMS (n = 36) MV ± SD	Difference MV (95% CI)	SMD (d`)	p-value
Visceral adipose tissue (VAT) volume [cm^3^]					
Baseline	569 ± 396	662 ± 382	------------	------	0.425
Changes	13 ± 66^n.s^	−16 ± 69^n.s^	28 (−5 to 61)	0.43	0.090

Applying ANCOVA that adjusted for baseline VAT and gender revealed significant gender differences with significantly higher WB-EMS-induced effects in men than in women (p = 0.032, d’ = 0.67). A separate analysis of the WB-EMS effects on VAT in women and men is provided in Table 3.

**TABLE 3:** Baseline data and changes in visceral adipose tissue parameters in the CG and WB-EMS groups categorized by gender. SMD: standardized mean difference; ^n.s.^: non-significant intra-group changes

Visceral adipose tissue (VAT) volume [cm^3^]					
Women	CG (n = 21) MV ± SD	WB-EMS (n = 22) MV ± SD	Difference MV (95% CI)	SMD (d`)	p-value
Baseline	493 ± 349	567 ± 300	------------	------	0.460
Changes	5 ± 45^n.s^	2 ± 47^n.s^	3 (−26 to 28)	0.07	0.911

Men	CG (n = 11)	WB-EMS (n = 14)			
Baseline	712 ± 396	813 ± 452	------------	------	0.581
Changes	29 ± 88^n.s^	−47 ± 92^n.s^	76 (−1 to 152)	0.83	0.052

In summary, although borderline non-significant (p = 0.052), we observed pronounced intra-group (WB-EMS vs CG) differences in men, while the effect of WB-EMS on VAT changes was negligible in women (p = 0.911).

### 3.2 Confounding factors

After the intervention period, no significant changes within the groups or between-group differences in physical activity (p = 0.124), exercise participation ≥60 min/w (p = 0.607), or exercise volume (p = 0.976) were reported by the participants. Five participants in the CG (three women and two men) and four participants in the WB-EMS (two women and two men) group (p = 0.794) reported changes in dietary habits, consistent with a reduction in carbohydrates/sugar and/or lower energy intake during the study period. Finally, no relevant changes in medication (e.g., glucocorticoids), conditions (e.g., eating disorders), or diseases (e.g., thyroid function) with potential impacts on abdominal adipose tissue parameters were reported.

## 4 Discussion

In this trial, which compared WB-EMS to a control group of standard care (physiotherapy), we could not find significant effects on VAT after 7 months of 1.5 × 20 min/week WB-EMS in overweight-to-obese adults. Reviewing the mechanisms of WB-EMS effects on body fat changes induced by increments of energy expenditure, largely comparable to conventional RT exercise, at least three effects can be identified. (a) First, acute energy expenditure during WB-EMS (Kemmler et al., 2012) is limited by the low training volume. (b) The post-exercise effect induced by energy restoration, repair, and adaptive processes post-exercise (EPOC) is particularly pronounced after WB-EMS application (Teschler et al., 2018). (c) Changes in the resting metabolic rate due to hypertrophic effects after WB-EMS (Kemmler et al., 2021).

A review of the literature shows that vigorous aerobic exercise/HIIT is particularly effective in reducing VAT in overweight-to-obese individuals (Chang et al., 2021; Chen et al., 2023; Poon et al., 2024). Aerobic exercise with low or moderate intensity, RT, or a combination of aerobic exercise and RT, on the other hand, is considered less favorable (Chang et al., 2021; Chen et al., 2023). Although there are a few exceptions (Chang et al., 2021), most meta-analyses (Chen et al., 2023; Khalafi et al., 2021; Lopez et al., 2022) reported significant (low to moderate) effect sizes for RT studies; single RCTs in the area of isolated RT and VAT rarely revealed significant effects. Thus, our results of positive, albeit non-significant findings of WB-EMS effects on VAT do not come as a surprise.

Nevertheless, a gender-specific sub-analysis of our data revealed differences with significantly more favorable, albeit still non-significant, VAT effects (WB-EMS vs CG) for the male subgroup (Tab. 3). This finding was supported by a recent network meta-analysis (Chen et al., 2023), which observed a much more pronounced VAT response to RT in men than in women. The same network meta-analysis (Chen et al., 2023) reported that in contrast to people with low body fat, RT was ineffective in reducing VAT in people with body fat rates ≥40%^1^. However, while only one man in the CG suffered from severe obesity, ten of the 22 women in the WB-EMS group had body fat rates ≥40%. In contrast to our finding of negligible WB-EMS effects on VAT in women, the pilot study by Park et al. (2021) reported significant positive effects on intra-abdominal fat after 8 weeks of 3 × 40 min/week of WB-EMS superimposed on aerobic dance in 30 older women with a similar body fat rate (39% ± 3%) compared to our female subgroup. Thus, there is some evidence that women need a higher training volume or/and superimposed WB-EMS to significantly affect the VAT. In contrast, a lower training volume seems to be sufficient for men for generating relevant VAT effects. This latter speculation was confirmed by a recent low-volume (2 × 40 min/week) RT trial with 43 older, predominantly overweight-to-obese men that determined similarly pronounced and (presumably due to the higher statistical power) significant net effects on VAT compared to the present male cohort (Knauer et al., 2023). In summary, adding significantly higher training frequency and strenuous voluntary exercises would not be a reasonable option, considering that the unique selling point of present WB-EMS concepts is their time-effective and joint-friendly characteristics.

Apart from cohort and exercise characteristics, one may argue that changes in dietary intake may have confounded the VAT results. Although participants were frequently instructed to maintain their dietary habits, nine of the 68 participants listed changes during the 29-week study period. As a similar number of people in the WB-EMS and CG groups reported changes in dietary habits, we feel that this aspect does not relevantly impact our findings. Undoubtedly, however, a more detailed monitoring of dietary records would have provided deeper insight into this issue.

Other limitations and peculiarities of the present trial of VAT should be addressed. (a) First, the primary outcome of this project, which included overweight and obese knee OA patients, was “pain of the knee joint”, while VAT was considered a secondary study outcome. One may argue that the alignment of the intervention was thus not customized to (optimally) address the VAT. However, the most effective WB-EMS protocol to achieve this outcome remains clear. Considering further that time efficiency is a key characteristic of WB-EMS, we applied the 20-min WB-EMS standard protocol (Beier et al., 2024; Le et al., 2024), which is known to trigger significant positive effects on a large variety of different outcomes (Kemmler et al., 2024). Another limitation of this satellite study of a larger project was the sample size analysis that did not address VAT. (b) Similarly, we did not focus on one gender but included women and men. Although the sample size of at least 32 participants/group included in the primary ITT analysis exceeds the statistical power of most RT studies with significant positive effects on VAT (Chen et al., 2023; Khalafi et al., 2021), in particular the low statistical power among the male subgroup (WB-EMS: n = 14 vs CG: n = 11) might have contributed to the borderline non-significant WB-EMS effect on VAT in men. (c) The study included adults with moderate-to-advanced knee osteoarthritis who were overweight and obese. Overweight/obesity is a strong predictor of knee OA (King et al., 2013), not only due to the higher mechanical load but also to the pro-inflammatory effects, particularly triggered by the VAT fraction (Kawai et al., 2021). Although the low-frequency, non-superimposed WB-EMS concepts might not be the most promising exercise for VAT reduction, the rationale for WB-EMS application in this cohort with OA was the opportunity for a joint-friendly protocol with low-amplitude, low-intensity movements/exercises. (d) Finally, another methodological weakness could be the lack of participant blinding. Optionally, the CG could have received the same intervention but with a pulse intensity below the motor threshold. We decided against this alternative since (a) even low stimulus intensity (e.g., low-intensity TENS) might trigger favorable effects on (knee) pain intensity (as the primary study outcome), and (b) we considered it more appropriate to provide a real-world comparison with an established therapy approach for knee OA.

In summary, we failed to determine significant positive effects of low-volume, non-superimposed WB-EMS on VAT, whether in women or (borderline) in men. Nevertheless, we are still convinced that individuals with OA will benefit from WB-EMS by improving their musculoskeletal outcomes. Although some participant characteristics might have contributed to our results, we conclude that, largely due to the low application volume, the present WB-EMS protocols applied most in scientific and commercial settings might not be the perfect tool for decreasing VAT, particularly in overweight-to-obese women.

## Data Availability

The raw data supporting the conclusions of this article will be made available by the authors without undue reservation.

## Footnotes

1 However, the author had not adjusted on gender differences. Overweight and obesity thresholds vary considerably between the genders (with lower values for men) when applying body fat rate (Tomlinson et al., 2019). Thus, gender differences might have confounded the effect of body fat rate (and *vice versa*) on VAT changes
